# Endocrinopathies in a Pediatric Patient Post-anatomical Hemispherectomy for Rasmussen’s Encephalitis Treatment: A Case Report

**DOI:** 10.7759/cureus.53894

**Published:** 2024-02-09

**Authors:** Jaron C Sanchez, Markeeta T Belmar, Jason C Sanchez, Kenny Nguygen

**Affiliations:** 1 Pediatrics, Nova Southeastern University Dr. Kiran C. Patel College of Osteopathic Medicine, Clearwater, USA; 2 Pediatrics, University of the Incarnate Word School of Osteopathic Medicine, San Antonio, USA; 3 Pediatrics, Bond Clinic, P.A., Winter Haven, USA

**Keywords:** hemispherotomy, central diabetes insipidus (cdi), central adrenal insufficiency, central precocious puberty, pediatric epileptic surgeries, endocrine abnormalities, endocrine disorder, pediatric seizure, hemispherectomy, rasmussen encephalitis

## Abstract

Hemispherectomy is a neurosurgical procedure that is frequently performed in pediatric patients diagnosed with Rasmussen's encephalitis. Postoperative complications include immediate complications such as hydrocephalus and hemorrhage and behavioral complications such as language impairments and contralateral weakness. However, there are limited studies or case reports that address the potential endocrinopathies associated with this and other pediatric epileptic surgeries. This case report describes the endocrinopathies following an anatomical hemispherectomy procedure.

A four-year-old African-American female had a right anatomical hemispherectomy for the treatment of Rasmussen's encephalitis in 2020. The postoperative course was immediately complicated by central diabetes insipidus which was stabilized with desmopressin. The patient’s labs in 2021 were consistent with central precocious puberty with elevated luteinizing hormone (LH) and follicle-stimulating hormone (FSH). Additionally, the patient was found to have secondary adrenal insufficiency in which she failed a low-dose adrenocorticotropic hormone (ACTH) stimulation test. Oral hydrocortisone therapy was initiated for secondary adrenal insufficiency in addition to initiating leuprolide injections for central precocious puberty. Furthermore, at the age of seven years, the patient had her first menarche.

This case report emphasizes the need for closer and long-term surveillance for endocrine issues in postepileptic surgical pediatric patients as well as a surveillance plan for the development of other potential endocrine abnormalities throughout the patient’s life.

## Introduction

Hemispherectomy is a radical neurosurgical procedure in which the diseased cerebral hemisphere is completely or partially removed or disconnected from the normal hemisphere. It is used to treat refractory or drug-resistant seizures and is frequently performed in pediatric patients diagnosed with Rasmussen's encephalitis [1]. Postoperative complications include but are not limited to cerebral edema, brain herniation, acute-onset hydrocephalus, hemorrhage, and electrolyte imbalances [2]. Additionally, long-term complications include but are not limited to impairments in reading, ambulation, spoken language, contralateral weakness, and cognitive disorders [3,4]. Most studies address the immediate and behavioral functions following this and other pediatric epileptic surgeries. However, there are limited studies or case reports that address the potential endocrinopathies associated with these procedures [5,6]. This case report looks into an African-American female pediatric patient with three different endocrine disorders, which are central diabetes insipidus, central precocious puberty, and secondary adrenal insufficiency, after undergoing a right anatomical hemispherectomy intervention for Rasmussen's encephalitis.

## Case presentation

We present a case of a four-year and two-month-old African-American female who had a right anatomical hemispherectomy for treatment of Rasmussen's encephalitis in May 2020. The patient has a known history of intractable seizures localized to the right cerebral hemisphere, left-sided hemiplegia in both upper and lower extremities, and left-eye esotropia. Additionally, the patient’s birth history was significant for a preterm birth at 29 weeks gestational age. The postoperative hemispherectomy was complicated by hypernatremia due to central diabetes insipidus and, upon discharge, her sodium levels in June stabilized to 144 mmol/L (reference range: 135-145 mmol/L) (Table 1) with desmopressin. However, the patient was readmitted a couple of weeks later to the hospital for hypernatremia with a serum level of 170 mmol/L. The patient’s sodium levels continued to be elevated and uncontrolled with serum levels of 167 mmol/L in December 2020, 156 mmol/L in May 2021, and 162 mmol/L in July 2021 which subsequently led to multiple hospital admissions. The patient’s persistent hypernatremia, as a result of the parents' lack of compliance with the medication regimen and fluid intake, continued to be an issue in several future clinic visits.

**Table 1:** Sodium levels

Electrolyte	June 2020	June 2020	December 2020	May 2021	July 2021	Reference Range
Age	4 yr 3 m	4 yr 3 m	4 yr 9 m	5 yr 2 m	5 yr 4 m	-
Na+	144	170	167	156	162	135-145 mmol/mL

Moreover, the patient’s labs in July 2021, at the age of five years and four months, were consistent with central precocious puberty with elevated luteinizing hormone (LH) of 2.2 mlU/mL (reference range: <0.2 mlU/mL) and follicle-stimulating hormone (FSH) of 5.9 mlU/mL (0.0-5.0 mlU/mL) (Table 2). Leuprolide therapy, a gonadotropin-releasing hormone agonist, was discussed with the father and an MRI of the pituitary gland was ordered. However, the MRI was never immediately followed up. Additionally, during a previous hospital admission in September 2021, the patient had secondary adrenal insufficiency as evident in an inadequate adrenocorticotropic hormone (ACTH) stimulation test. Oral hydrocortisone therapy was initiated for secondary adrenal insufficiency in addition to initiating leuprolide injections for the central precocious puberty. Following the hospitalization, the patient was lost to follow-up for one year but returned to the clinic in August 2022. It was revealed that the father had missed several endocrinology appointments and was unsure when was the patient’s last leuprolide injection. In addition, during the August 2022 visit when the patient's calendar age was six years and five months, the patient’s physical exam was significant for Tanner stage one for pubic hair development and Tanner stage two for breast development since the patient displayed no pubic hair development and had nipple protrusion with a slight increase in areolar diameter.

**Table 2:** Hormone levels in July 2021 and June 2023 LH: luteinizing hormone, FSH: follicle-stimulating hormone, DHEA: dehydroepiandrosterone, TSH: thyroid-stimulating hormone, T4: thyroxine

Hormone	July 2021	June 2023	Reference Range
Age	5 yr 4 m	7 yr 3 m	-
LH	2.2	3.1	<0.2 mlU/mL
FSH	5.9	4.7	0.0-5.0 mlU/mL
DHEA-Sulfate (serum)	-	75.4	26.1-141.9 ug/dL
TSH	-	3.1	0.66-4.75 ulU/mL
Free T4	-	1.00	0.91-1.45 ng/dL
Prolactin	-	5.8	5.0-20 ng/mL
Progesterone	-	0.4	≤0.35 ng/mL

Furthermore, in June 2023 at the age of seven years and three months, the patient had her first menarche according to the grandmother. Consequently, according to the reference atlas of Greulich and Pyle, which exhibited skeletal maturation at different ages, the skeletal bone age for this patient was determined to be 10 years. Labs during this visit demonstrated elevated LH (serum: 3.1 mlU/mL; reference range: <0.2 mlU/mL) and progesterone (serum: 0.4 ng/mL; reference range: ≤0.35 ng/mL) with normal FSH (serum: 4.7 mlU/mL; reference range: 0.0-5.0 mlU/mL), dehydroepiandrosterone sulfate (serum: 75.4 ug/dL; reference range: 26.1-141.9 ug/dL), thyroid-stimulating hormone (serum: 3.1 ulU/mL; reference range: 0.66-4.75 ulU/mL), free thyroxine (serum: 1.00 ng/dL; reference range: 0.91-1.45 ng/dL), and prolactin (serum: 5.8 ng/mL; reference range: 5.0-20 ng/mL) (Table 2). Additionally, the patient’s physical exam was significant for Tanner stage four for both pubic hair and breast development since the patient displayed adult pubic hair that does not extend to the inner thighs and had nipple and areola forming a secondary mound projecting above the breast tissue. Upon follow-up with pediatric neurosurgery, the brain MRI in June 2023 revealed stable, mild ventriculomegaly, atrophic changes, axonal degeneration of the right cerebral peduncle and hemisphere, mild decrease in size of the left cerebellar folia, and normal morphology of the hypothalamic-pituitary axis. The patient had been seizure-free since the surgery.

## Discussion

Rasmussen's encephalitis is a rare chronic neurological disorder of unknown origin characterized by inflammation affecting the cerebral cortex of one hemisphere and is typically resistant to antiepileptic medications [1]. The median age of onset is six years but can range from infancy to adulthood [1]. In the view that Rasmussen's encephalitis is an immune-regulated process, immunotherapy treatments such as long-term corticosteroids, intravenous immunoglobulins, and tacrolimus have been used to alleviate symptoms [7]. However, for patients with refractory symptoms, surgery remains the only cure with procedures that disconnect the affected hemisphere. Two such procedures are a hemispherectomy, which is the complete removal of the hemisphere, or hemispherotomy, which is the focal resection of the hemisphere [8]. Postoperative complications include but are not limited to cerebral edema, brain herniation, acute-onset hydrocephalus, hemorrhage, and electrolyte imbalances [2]. Additionally, long-term complications include but are not limited to impairments in reading, ambulation, spoken language, contralateral weakness, and cognitive disorders [3,4].

However, there are limited studies or case reports that address the potential endocrinopathies associated with these procedures. A retrospective study by Saito et al. identified patients with postoperative central diabetes insipidus as a sequela of hemispherotomy or hemispherectomy in infants who were less than 12 months old. However, Saito et al. did not investigate or report other possible endocrine sequelae [6]. Additionally, a retrospective study published in 2020 by Jones et al. investigated postepileptic surgeries in patients who were less than 18 years of age and found that a higher incidence of precocious puberty and diabetes insipidus were seen in children post-hemispherectomy. This study also emphasized the need for closer and long-term surveillance following pediatric epileptic surgeries to better understand the true prevalence and etiology of these endocrinopathies [5]. Our patient presented above had three endocrine disorders which were central diabetes insipidus, central precocious puberty, and secondary adrenal insufficiency following a right anatomical hemispherectomy. It was interesting to note secondary adrenal insufficiency was diagnosed in our patient one year after the surgery. This further emphasizes the need for closer and long-term surveillance of this patient population.

Furthermore, endocrinopathies following a hemispherectomy aren’t common or at least not well documented compared to other brain surgeries such as transsphenoidal hypophysectomy, which postoperatively can be complicated by hypopituitarism or diabetes insipidus [9-11]. However, due to the nature of a hemispherectomy procedure, there could be a possibility of affecting structures or pathways related to the endocrine system such as the hypothalamus or pituitary gland [12,13]. Studies have shown that the postoperative course of children following brain surgeries is commonly complicated by hydrocephalus. Consequently, these children are also prone to endocrinological problems such as precocious puberty, short stature, or amenorrhea due to the harmful cerebrospinal fluid pressure on the brain’s tissue [14,15]. In a hemispherectomy procedure, obstructive hydrocephalus can arise postoperatively possibly affecting the hypothalamus, pituitary gland, or other endocrine structures [2]. However, in our patient, obstructive hydrocephalus was absent, but instead had mild stable ventriculomegaly. Although an MRI of the pituitary was never performed following the diagnosis of central precocious puberty in 2021, the latest brain MRI in 2023 revealed stable, mild ventriculomegaly, atrophic changes, axonal degeneration of the right cerebral peduncle and hemisphere, mild decrease in size of the left cerebellar folia, and normal morphology of the hypothalamic-pituitary axis. To this end, there could be undetectable microtrauma, stress, or possibly the pressure from the mild ventriculomegaly which could be affecting the structures in the hypothalamic-pituitary axis and thereby causing further endocrine abnormalities.

## Conclusions

Pediatric patients undergoing pediatric epileptic surgeries such as hemispherectomy or hemispherotomy may have postoperative endocrine complications in addition to other immediate or delayed complications. Among the endocrinopathies, it commonly includes central diabetes insipidus and central precocious puberty as well as secondary adrenal insufficiency. This case report emphasizes the need for closer and long-term surveillance for endocrine issues in postepileptic surgical pediatric patients as well as a surveillance plan for the development of other potential endocrine abnormalities throughout the patient’s life.

## References

[1] Varadkar S, Bien CG, Kruse CA (2014). Rasmussen's encephalitis: clinical features, pathobiology, and treatment advances. Lancet Neurol.

[2] Brotis AG, Georgiadis I, Fountas KN (2015). Hemispherectomy: indications, surgical techniques, complications, and outcome. J Neurol and Neurophysiol.

[3] Moosa AN, Jehi L, Marashly A (2013). Long-term functional outcomes and their predictors after hemispherectomy in 115 children. Epilepsia.

[4] Borne A, Perrone-Bertolotti M, Jambaqué I (2022). Cognitive outcome after left functional hemispherectomy on dominant hemisphere in patients with Rasmussen encephalitis: beyond the myth of aphasia. Patient series. J Neurosurg Case Lessons.

[5] Jones M, Zeitler PS (2020). SUN-057 Endocrine dysfunction after pediatric epilepsy surgery: a report from the global pediatric epilepsy surgery registry. J Endocr Soc.

[6] Saito T, Sugai K, Takahashi A (2020). Transient water-electrolyte disturbance after hemispherotomy in young infants with epileptic encephalopathy. Childs Nerv Syst.

[7] Lagarde S, Boucraut J, Bartolomei F (2022). Medical treatment of Rasmussen's encephalitis: a systematic review. Rev Neurol (Paris).

[8] Kim JS, Park EK, Shim KW, Kim DS (2018). Hemispherotomy and functional hemispherectomy: indications and outcomes. J Epilepsy Res.

[9] Zubair A, M Das J (2023). Transsphenoidal hypophysectomy. StatPearls [Internet].

[10] Alzhrani G, Sivakumar W, Park MS, Taussky P, Couldwell WT (2018). Delayed complications after transsphenoidal surgery for pituitary adenomas. World Neurosurg.

[11] Ausiello JC, Bruce JN, Freda PU (2008). Postoperative assessment of the patient after transsphenoidal pituitary surgery. Pituitary.

[12] Vignolles-Jeong J, Kreatsoulas D, Godil S, Otto B, Carrau R, Prevedello D (2022). Complications in endoscopic pituitary surgery. Otolaryngol Clin North Am.

[13] Lee JY, Lee YA, Jung HW (2017). Long-term endocrine outcome of suprasellar arachnoid cysts. J Neurosurg Pediatr.

[14] Bereket A (2019). Growth and puberty in hydrocephalus. Pediatric Hydrocephalus.

[15] Pinto FC, da Cunha Neto MB, Rocha MG, do Lago DV, Bronstein MD, Teixeira MJ (2011). Hypopituitarism due to hydrocephalus: case report and review of the literature. Pediatr Neurosurg.

